# Artificial Intelligence–Based Psoriasis Severity Assessment: Real-world Study and Application

**DOI:** 10.2196/44932

**Published:** 2023-03-16

**Authors:** Kai Huang, Xian Wu, Yixin Li, Chengzhi Lv, Yangtian Yan, Zhe Wu, Mi Zhang, Weihong Huang, Zixi Jiang, Kun Hu, Mingjia Li, Juan Su, Wu Zhu, Fangfang Li, Mingliang Chen, Jing Chen, Yongjian Li, Mei Zeng, Jianjian Zhu, Duling Cao, Xing Huang, Lei Huang, Xing Hu, Zeyu Chen, Jian Kang, Lei Yuan, Chengji Huang, Rui Guo, Alexander Navarini, Yehong Kuang, Xiang Chen, Shuang Zhao

**Affiliations:** 1 Department of Dermatology Xiangya Hospital Central South University Changsha, Hunan China; 2 National Engineering Research Center of Personalized Diagnostic and Therapeutic Technology Hunan China; 3 Hunan Key Laboratory of Skin Cancer and Psoriasis Xiangya Hospital Central South University Hunan China; 4 National Clinical Research Center for Geriatric Disorders Xiangya Hospital Central South University Hunan China; 5 Tencent Beijing China; 6 Department of Dermatology Dalian Dermatosis Hospital Liaoning China; 7 Mobile Health Ministry of Education - China Mobile Joint Laboratory Xiangya Hospital Central South University Changsha, Hunan China; 8 Department of Dermatology Second Affiliated Hospital of Nanhua University Hengyang, Hunan China; 9 Department of Dermatology Shaoyang Central Hospital Shaoyang, Hunan China; 10 Department of Dermatovenerology The First People's Hospital Of Changde City Changde, Hunan China; 11 Department of Dermatology Xiangtan Central Hospital Xiangtan, Hunan China; 12 Department of Dermatology Hunan Provincial People's Hospital (The First Affiliated Hospital of Hunan Normal University) Changsha, Hunan China; 13 State Key Laboratory of High Performance Complex Manufacturing College of Mechanical and Electrical Engineering Central South University Changsha, Hunan China; 14 Department of Dermatology Third Xiangya Hospital Central South University Changsha, Hunan China; 15 China Mobile (Chengdu) Industrial Research Institute Chengdu, Sichuan China; 16 Department of Dermatology University Hospital of Basel Basel Switzerland; 17 Department of Biomedical Engineering University Hospital of Basel Basel Switzerland

**Keywords:** artificial intelligence, psoriasis severity assessment, Psoriasis Area and Severity Index, PASI, deep learning system, mobile app, psoriasis, inflammation, dermatology, tools, management, model, design, users, chronic disease

## Abstract

**Background:**

Psoriasis is one of the most frequent inflammatory skin conditions and could be treated via tele-dermatology, provided that the current lack of reliable tools for objective severity assessments is overcome. Psoriasis Area and Severity Index (PASI) has a prominent level of subjectivity and is rarely used in real practice, although it is the most widely accepted metric for measuring psoriasis severity currently.

**Objective:**

This study aimed to develop an image–artificial intelligence (AI)–based validated system for severity assessment with the explicit intention of facilitating long-term management of patients with psoriasis.

**Methods:**

A deep learning system was trained to estimate the PASI score by using 14,096 images from 2367 patients with psoriasis. We used 1962 patients from January 2015 to April 2021 to train the model and the other 405 patients from May 2021 to July 2021 to validate it. A multiview feature enhancement block was designed to combine vision features from different perspectives to better simulate the visual diagnostic method in clinical practice. A classification header along with a regression header was simultaneously applied to generate PASI scores, and an extra cross-teacher header after these 2 headers was designed to revise their output. The mean average error (MAE) was used as the metric to evaluate the accuracy of the predicted PASI score. By making the model minimize the MAE value, the model becomes closer to the target value. Then, the proposed model was compared with 43 experienced dermatologists. Finally, the proposed model was deployed into an app named SkinTeller on the WeChat platform.

**Results:**

The proposed image-AI–based PASI-estimating model outperformed the average performance of 43 experienced dermatologists with a 33.2% performance gain in the overall PASI score. The model achieved the smallest MAE of 2.05 at 3 input images by the ablation experiment. In other words, for the task of psoriasis severity assessment, the severity score predicted by our model was close to the PASI score diagnosed by experienced dermatologists. The SkinTeller app has been used 3369 times for PASI scoring in 1497 patients from 18 hospitals, and its excellent performance was confirmed by a feedback survey of 43 dermatologist users.

**Conclusions:**

An image-AI–based psoriasis severity assessment model has been proposed to automatically calculate PASI scores in an efficient, objective, and accurate manner. The SkinTeller app may be a promising alternative for dermatologists’ accurate assessment in the real world and chronic disease self-management in patients with psoriasis.

## Introduction

Psoriasis is a chronic, immune-mediated disease that causes pain, disfigurement, and disability, for which there is no cure, as recognized by the World Health Organization [1]. It is characterized by erythematous plaques covered by white scales, which are occasionally pruritogenic, and affects approximately 125 million people worldwide [2]. Psoriasis has posed heavy burden to global economics, including the increased cost of treatment, functional impairment, and loss of career opportunities [3]. Precision therapy and long-term management are required to reduce the risk of comorbidities, inflammatory arthritis, cardiometabolic diseases, and mental disorders and to avoid culminating in a reduced life span [4-6]. The treatment and management plan of psoriasis is usually established according to the degree of psoriasis severity. Hence, a comprehensive, accurate, and objective assessment tool of psoriasis severity is necessary for a personalized approach to diagnose and treat patients.

Psoriasis Area and Severity Index (PASI) is the most widely accepted metric for measuring psoriasis severity currently [7]. A total PASI score (range 0-72), with higher scores indicating more severity, is determined by visual inspection of skin area (0-6), erythema (0-4), desquamation (0-4), and induration (0-4) of 4 body parts: head, trunk, upper limbs, and lower limbs (details can be seen in Multimedia Appendix 1). However, PASI is known to have a prominent level of subjectivity and poor intra- and interobserver consistency [8,9], although it is informative and reasonably useful to evaluate psoriasis severity. Our survey of 346 dermatologists from different hospitals throughout China found PASI scoring to be challenging in daily routine clinics (Multimedia Appendix 2). Only 30.9% (n=107) of physicians preferred to evaluate the severity of psoriasis by PASI in clinical practice. Therefore, the PASI method needs to be optimized to become more objective, intelligent, and convenient in the real world.

Deep neural models have been proposed for diagnosing and evaluating multiple skin diseases [10]. Several studies have explored the related applications of artificial intelligence (AI) in psoriasis [11-14] but focused mainly on the assisted diagnosis. As for psoriasis severity rating, only a few attempts have been conducted. Existing works either only calculate a subscore of PASI rather than the full PASI score or are limited to the offline evaluation while lacking the comparison with experienced dermatologists and collection of feedback from clinical application [15-18].

In this study, we proposed a deep learning system based on a psoriasis-specific image database and developed a mobile app (named SkinTeller) integrated with this model to assess psoriasis severity in clinical practice. Results from multiple centers highlight the potential to achieve precise treatment and management for psoriasis.

## Methods

### Ethics Approval

This study was approved by the institutional Clinical Research Ethics Committee of Xiangya Hospital (No.2021101068).

### Participants

This study collected information about consecutive multicenter patients with psoriasis aged 18 years and older from January 2015 to July 2021.

### Data Collection

A data set of 14,096 images from 2367 patients with psoriasis taken by digital cameras or smartphones was established for this study. We used 1962 patients from January 2015 to April 2021 to train the model and the other 405 patients from May 2021 to July 2021 to validate it. All patients gave their informed consent before collecting images. Each image was captured with either a digital camera (SONY DSC-HX50) or a smartphone (Apple iPhone 6s to 12). Three clinicians who have more than 10 years of experience in assessing psoriasis severity were invited to label the images independently. The final PASI scores of each image were obtained from the average of their scores, which can be viewed as ground truth labeling. To further ensure the reliability of the data, each data point was also checked by at least one professor-level doctor. Considering the images were taken under different lighting conditions, we normalized the input images to ensure the generalizability of the model. An offline automatic color enhancement algorithm [19] was implemented to reduce the influence of varied color and luminance conditions.

### Model Development

**Figure 1:**
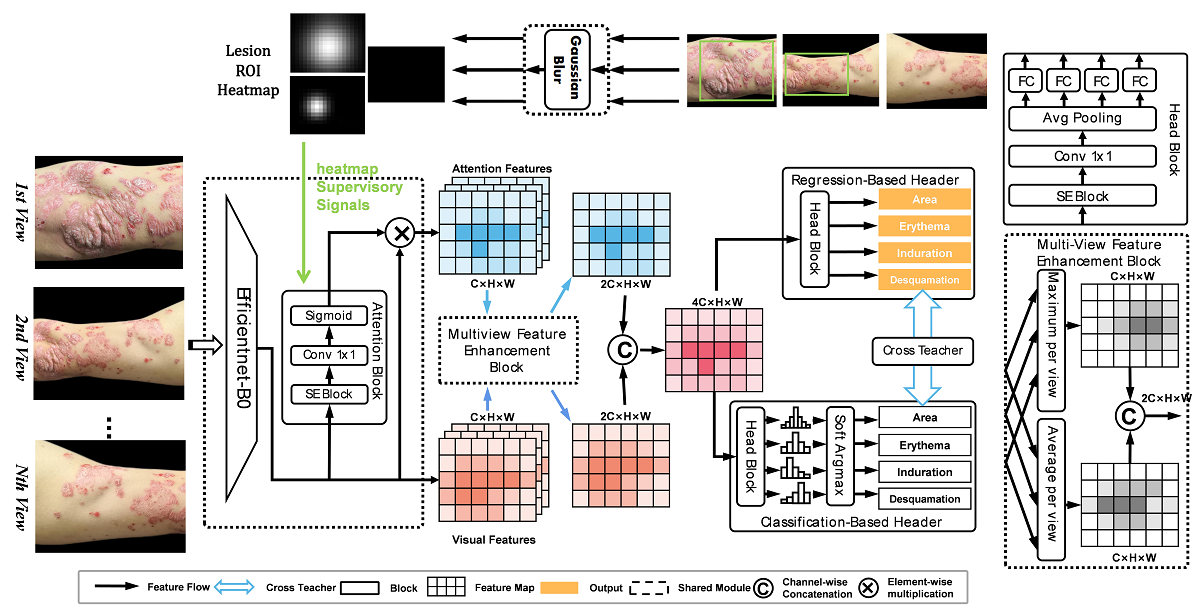
The structure of the proposed image–artificial intelligence (AI)–based Psoriasis Area and Severity Index (PASI) assessment model, which refers to the PASI score rating module. Avg: average; C: channel; conv: convolution; FC: fully connected; H: height; ROI: region of interest; SE: squeeze and excitation; W: width.

A severity assessment model was proposed to simulate how dermatologists calculate PASI scores in the clinical practice (Figure 1). The model receives N lesion images from each body part as inputs and predicts the redness of erythema (0-4), the thickness of induration (0-4), the scaling of desquamation (0-4), and the area ratio (0-6).

This model consists of an image-processing block, a multiview feature enhancement block, and a cross-revise output block. During the training stage, the image-processing block first conducts random cropping of the input image and then resizes the cropped image to 800 × 1024 pixels. Then, a convolutional neural network (CNN) encoder is used to extract visual features from the processed image, in which another attention branch is specially designed to generate attention features for the regions of interest (ROIs). In this task, ROIs denote the regions with skin lesions in the input images. Afterward, the visual feature and the attention feature are combined as the final feature representation, in which the features unrelated to ROIs are suppressed. In this study, a basic EfficientNet-B0 model was applied as the CNN encoder, and 2143 images containing manually skin lesions marked by bounding boxes served as the supervisory signals to the attention branch in the heat map mask format.

Furthermore, a multiview feature enhancement block that merges features from multiple input images was designed to enable the model to combine vision features from different perspectives. Specifically, this block handles the visual and attention features by calculating their maximum and average values to convert these image-level features into patient-level features. Then, the multihead output block uses the obtained patient-level features as input and sends them into 2 output headers with different optimization objectives, that is, a regression header with a smooth L1 loss and a classification header with a softmax cross-entropy loss. Moreover, we designed an extra cross-teacher loss [20] that revises the output of either output header by the other headers to learn more prior knowledge from the severity score annotated by dermatologists. Finally, the regression output is used as the final output. This may simulate the assessment method used by dermatologists in real clinical practice.

### Model Validation

Patients who visited hospitals from May 2021 to July 2021 were included in a separate validation cohort, which was conducted as a prospective analysis.

The accuracy of the predicted PASI score was first evaluated. The mean average error (MAE) was used as the evaluation metric, which measures the average squared difference between the estimated values and the target values. In this study, we measured the absolute distance from the predicted score obtained from our model to the actual PASI score diagnosed by dermatologists. Besides the overall score, the accuracy of the 4 subscores of PASI was also evaluated. Erythema, induration, and desquamation are represented by integer values ranging from 0 to 4, and the area ratio is represented by an integer value ranging from 0 to 6. Therefore, we formulated the subscore predictions as 5-label and 7-label classifications, respectively, and used the classification accuracy as the evaluation metric. The trend predicted by the proposed model was further evaluated. If the PASI score increases, it potentially indicates a deterioration of psoriasis. If the PASI score decreases, it potentially indicates an improvement of psoriasis.

### Comparison With Dermatologists

The proposed neural model was compared with 43 experienced dermatologists from 18 hospitals. Similar to the model validation, 2 perspectives revealed the comparison: (1) the accuracy of PASI scoring for each visit and (2) the severity ranking between 2 visits. To conduct a fair comparison, we provided the same skin lesion images that were not included in the training and testing data set to both the dermatologists and the proposed model. A labeling website was built for dermatologists to annotate the images on the web.

### Trend Prediction Validation

In all, 429 patients’ visits were followed up. Among them, 214 visits were of low severity (PASI≤5), 119 visits were of medium severity (5<PASI≤10), and 96 visits were of high severity (PASI>10). All pairs of these visits were enumerated, that is, C^2^_429_=91,806 pairs in total. For each pair, we predicted the PASI score of these 2 visits. If the order of the predicted PASI scores was the same as the order of manual annotated PASI scores, we regarded it as a successful trend prediction; otherwise, we regarded it as an incorrect prediction.

### App Deployment

We deployed the proposed severity assessment model into an app named SkinTeller on the WeChat platform. WeChat is a social network platform that is similar to WhatsApp and Line. WeChat processes 1.2 billion monthly active users [21]. Since WeChat allows users to launch mini-apps without installation or configuration, the app provides convenient access to the proposed model for both dermatologists and patients with psoriasis.

## Results

### Overview of the Workflow

**Figure 2:**
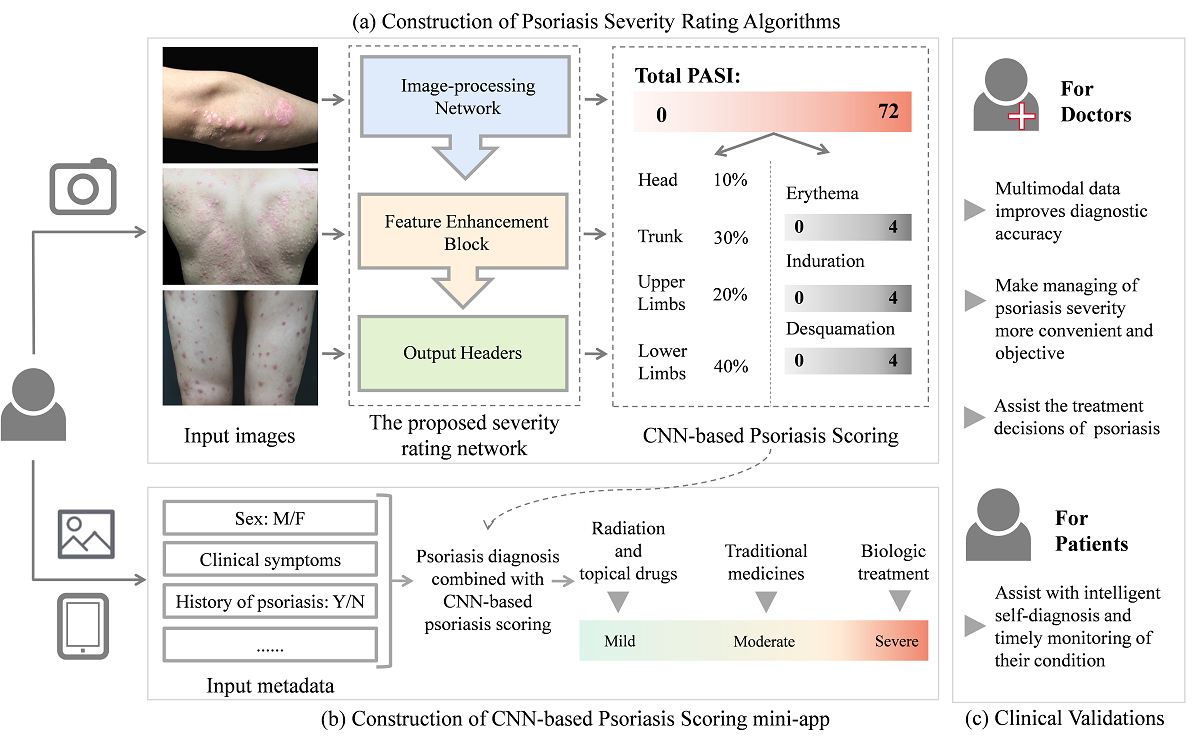
Overview of the workflow of the proposed model and the SkinTeller mobile app. (a) The image–artificial intelligence (AI)–based Psoriasis Area and Severity Index (PASI)–estimating model. (b) The workflow of the SkinTeller app that is integrated with the proposed model. (c) The clinical significance for both doctors and patients. CNN: convolutional neural network; F: female; M: male; N: no; Y: yes.

In this study, we integrated the proposed image-AI–based PASI-estimating model into the SkinTeller app and optimized the process of psoriasis severity assessment (Figure 2). Patients were allowed to take images of skin lesions and complete their medical history. The proposed model should output the PASI score with higher accuracy, and its consistency should outperform the actual clinical applications. We are also exploring making decisions based on psoriasis diagnosis and treatment guidelines and the experience of doctors and designing the product to automatically classify psoriasis severity into mild, moderate, and severe conditions based on the score and provide treatment recommendations. For example, patients with mild conditions should be treated with phototherapy and topical drugs, whereas patients with severe conditions should be recommended to biologic treatment. This work is important for both doctors and patients.

### Performance of the Model by Ablation Studies

Generally, with more photos, the model has more information about the skin condition and therefore is more likely to predict an accurate PASI score. As a result, the first ablation study was conducted on the number of input lesion images per body part. Table 1 illustrates the performance of the model with different numbers of input images. The MAE decreased from 3.47 to 2.25 when the number of input image increased from 1 to 2. When entering 3 images, the performance of our model reached the peak and achieved the smallest MAE of 2.05. By the first ablation study, the error of the model is at minimum when given 3 images. In other words, this situation is closer to the real PASI score. The next ablation study compared different fusion strategies for multiple images to reveal the performance of the multiview feature enhancement block. As shown in Table 1, the combination of multiple aggregation methods outperformed the methods individually, whether in the total score or the 4 subindicators of PASI. The third study evaluated the performance of different output headers: the classification header, the regression header, and the cross-teacher header. According to the definition of the PASI score, the values of erythema, induration, desquamation, and area ratio are all discrete integers. In this manner, we formulated severity rating as a classification task. However, considering the ordering of different values, the task can also be formulated as a regression problem. According to Table 1, the aggregation of both the regression header and the classification header with the cross-teacher header outperformed other approaches.

For different groups of patients with varied degrees of severity, the performance of the proposed model decreased when the degree of severity increased (Table 1). For patients with low and medium severity, the MAE of the overall PASI score was less than 2; for patients with high severity, the MAE enlarged to 3.29. This is because the PASI score includes multiplication operations between subscores. Since the values of these subscores are already very large, a small variation of one subscore could result in a big variation of the total PASI score.

**Table 1:** Ablation studies.

Item			Area ratio (%)		Erythema (%)		Desquamation (%)		Induration (%)		MAE^a^ of PASI^b^
**Number of input image**											
	1	63.7		59.29		53.64		55.65		3.47	
	2	70.69		63.41		56.23		62.55		2.25	
	3	*70.98* ^c^		*64.56*		57.76		*65.33*		*2.05*	
	4	69.16		56.23		*59*		62.55		2.08	
**Fusion strategy**											
	Only max	64.94		59.39		55.65		61.4		2.73	
	Only mean	69.83		62.16		53.26		61.49		2.31	
	Max + mean	*70.98*		*64.56*		*57.76*		*65.33*		*2.05*	
**Output method**											
	Only regression	68.87		61.49		57.95		60.82		2.14	
	Only classification	62.55		*64.85*		52.97		59.2		2.61	
	Regression + classification	69.73		63.89		*58.52*		63.31		2.21	
	Regression + classification with cross-teacher	*70.98*		64.56		57.76		*65.33*		*2.05*	
**Severity level (number of visits)**											
	All (N=429)	67.54		63.46		58.39		61.6		1.97	
	Mild (0-5; n=214)	70.27		59.1		53.64		59.1		1.70	
	Moderate (6-10; n=119)	66.10		67.61		66.67		58.33		1.48	
	Severe (>10; n=96)	63.46		67.31		57.14		71.98		3.29	

^a^MAE: mean absolute error.

^b^PASI: Psoriasis Area and Severity Ratio.

^c^Italicized values indicate the best performance.

### Comparison With Dermatologists

In all, 43 dermatologists from 18 hospitals participated and finished the labeling. They consisted of 13 professors (at least 15 years of dermatological experience) and 30 attending or resident physicians (at least 5 years of dermatological experience). As shown in Table 2, the proposed model outperformed dermatologists with a 33.2% improvement in the total PASI score and 23%, 7%, 11%, and 12% improvements in accuracy in the 4 subscores of erythema, induration, desquamation, and area ratio, respectively. The greatest improvement is erythema, which is also because it has the strongest subjectivity. The proposed model ranked eighth among all 43 dermatologists using MAE as the main measurement (dermatologist mean MAE=4.67; range 2.51-7.49; proposed model MAE=3.12). The comparison proved the effectiveness of the proposed severity rating model, considering that the majority of participant dermatologists are experienced in PASI scoring.

**Table 2:** Performance comparison of the deep learning system versus the dermatologists (professors and attending or resident physicians). The proposed model achieved better results than dermatologists in the 4 subscores of Psoriasis Area and Severity Index (PASI), including erythema, induration, desquamation, and area ratio.

	Erythema (%)	Induration (%)	Desquamation (%)	Area ratio (%)
Dermatologists (professors)	39	36	37	60
Dermatologists (attending or resident physicians)	43	44	43	50
Dermatologists (combined)	42	44	43	54
Proposed model	45	42	43	55

### Trend Prediction Validation

The accuracy in predicting the direction of severity progress with different ranges of PASI scores were further evaluated (Table 3). The average trend accuracy was 84.81%, indicating that the proposed model could accurately predict the trend of the severity degree for over 80% of cases. We also found that if the score gap between 2 visits is larger than 5 PASI score points, that is, if the severity degree changes from severe to moderate or from moderate to mild, we could predict the event at an accuracy level as high as 96.09%. However, the interagreement between dermatologists was poor. For top-ranked dermatologists, they tended to better agree with each other, whereas low-ranked dermatologists were more likely to disagree with each other (Multimedia Appendix 3).

**Table 3:** The consistent trend between artificial intelligence and doctors among different Psoriasis Area and Severity Index (PASI) score gaps.

PASI score gap	Trend consistency (%)
Average	84.81
>10	98.79
6-10	96.09
0-5	73.26

### Case Study

**Figure 3:**
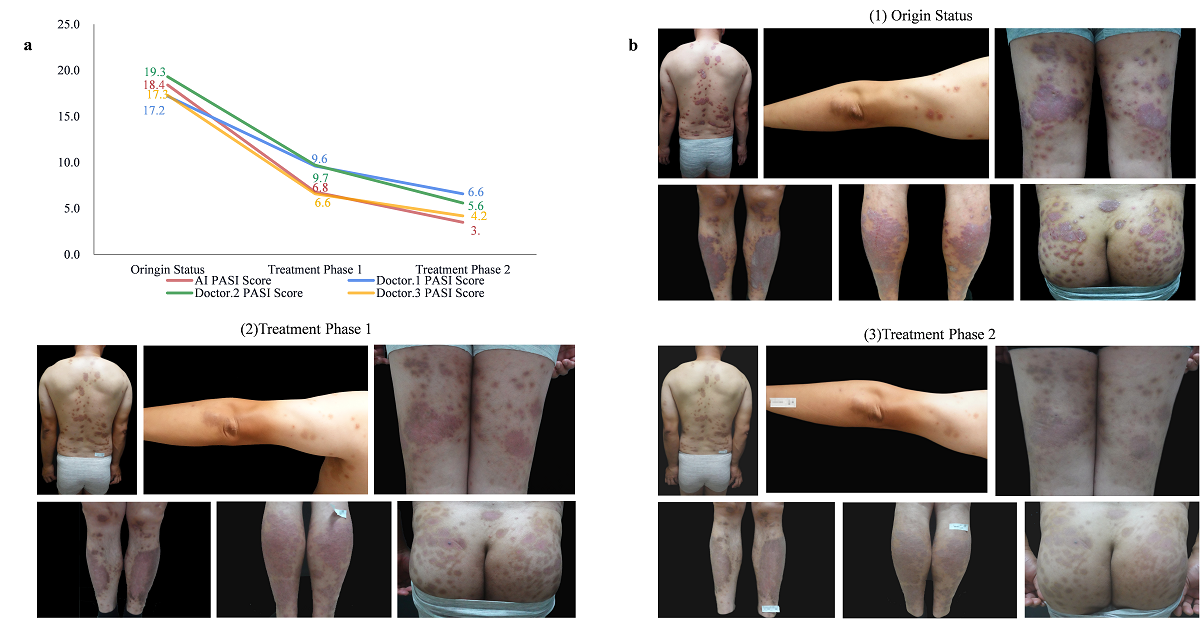
The practical application of the proposed image-AI–based PASI-estimating model. (a) The PASI scores between the AI and 3 doctors for a patient with psoriasis at different treatment phases. (b) The clinical images of the patient at different treatment phases. AI: artificial intelligence; PASI: Psoriasis Area and Severity Index.

A complete treatment course of a patient with psoriasis was tracked. Figure 3 shows the PASI scores of the proposed system and doctors at different treatment phases. The trend of the severity was generally consistent, whereas there were some small discrepancies in the scores from the AI and 3 doctors. The AI and all 3 doctors assigned psoriasis scores over 15 as the original status. Large areas of erythema and desquamation were observed all over the skin surface of the patient, and most of the subscores for the erythema metric were 3 or 4 (Figure 3 and Table 4). After the first treatment phase, the patient exhibited a substantial treatment response with the disappearance of lesions on the head with all scores being 0. The PASI score suggested that the treatment achieved substantial improvements in the erythema, induration, and desquamation of the patient’s lesions. However, due to the excessive size of the patient’s lesions, the PASI scores of the lower limbs assigned by the AI and all 3 doctors remained high after the first treatment phase, especially in the area ratio (PASI scores of 2, 3, 3, and 2, respectively) and erythema (PASI scores of 3, 3, 2, and 2, respectively). After 2 phases of treatment, the AI and all 3 doctors considered the lesions on the head and upper limbs to be completely relieved, which resulted in a PASI score of 0.

Similarly, we reported on another patient with psoriasis to present the PASI scores between AI and dermatologists during different treatment phases (Multimedia Appendix 4).

**Table 4:** The Psoriasis Area and Severity Index (PASI) scores between artificial intelligence (AI) and 3 doctors for a patient with psoriasis at different treatment phases.

			Area ratio	Erythema	Induration	Desquamation	PASI score	
**Original status**								
	**AI**							18.4
		Head	1	2	1	1		
		Trunk	2	3	2	3		
		Upper limb	1	3	1	2		
		Lower limb	3	4	3	3		
	**Doctor 1**							17.2
		Head	1	2	1	1		
		Trunk	2	3	2	3		
		Upper limb	1	3	1	2		
		Lower limb	3	3	3	3		
	**Doctor 2**							19.3
		Head	1	1	1	1		
		Trunk	2	3	1	2		
		Upper limb	1	2	1	2		
		Lower limb	4	3	3	3		
	**Doctor 3**							17.3
		Head	1	2	1	1		
		Trunk	2	3	1	3		
		Upper limb	1	2	1	1		
		Lower limb	3	4	3	3		
**Treatment phase 1**								
	**AI**							6.8
		Head	0	0	0	0		
		Trunk	2	2	1	1		
		Upper limb	1	1	0	1		
		Lower limb	2	3	1	1		
	**Doctor 1**							9.6
		Head	0	0	0	0		
		Trunk	2	2	1	1		
		Upper limb	0	0	0	0		
		Lower limb	3	3	1	2		
	**Doctor 2**							9.7
		Head	1	1	0	0		
		Trunk	2	1	1	1		
		Upper limb	1	1	1	1		
		Lower limb	3	2	2	2		
	**Doctor 3**							6.6
		Head	0	0	0	0		
		Trunk	2	1	1	1		
		Upper limb	1	2	1	1		
		Lower limb	2	2	1	2		
**Treatment phase 2**								
	**AI**							3.5
		Head	0	0	0	0		
		Trunk	3	3	2	3		
		Upper limb	0	0	0	0		
		Lower limb	2	2	1	1		
	**Doctor 1**							6.6
		Head	0	0	0	0		
		Trunk	2	2	1	2		
		Upper limb	0	0	0	0		
		Lower limb	3	1	1	1		
	**Doctor 2**							5.6
		Head	0	0	0	0		
		Trunk	2	1	1	2		
		Upper limb	0	0	0	0		
		Lower limb	2	2	1	1		
	**Doctor 3**							4.2
		Head	0	0	0	0		
		Trunk	2	2	1	1		
		Upper limb	0	0	0	0		
		Lower limb	2	1	1	1		

### Functions and Users’ Feedback of the SkinTeller App

**Figure 4:**
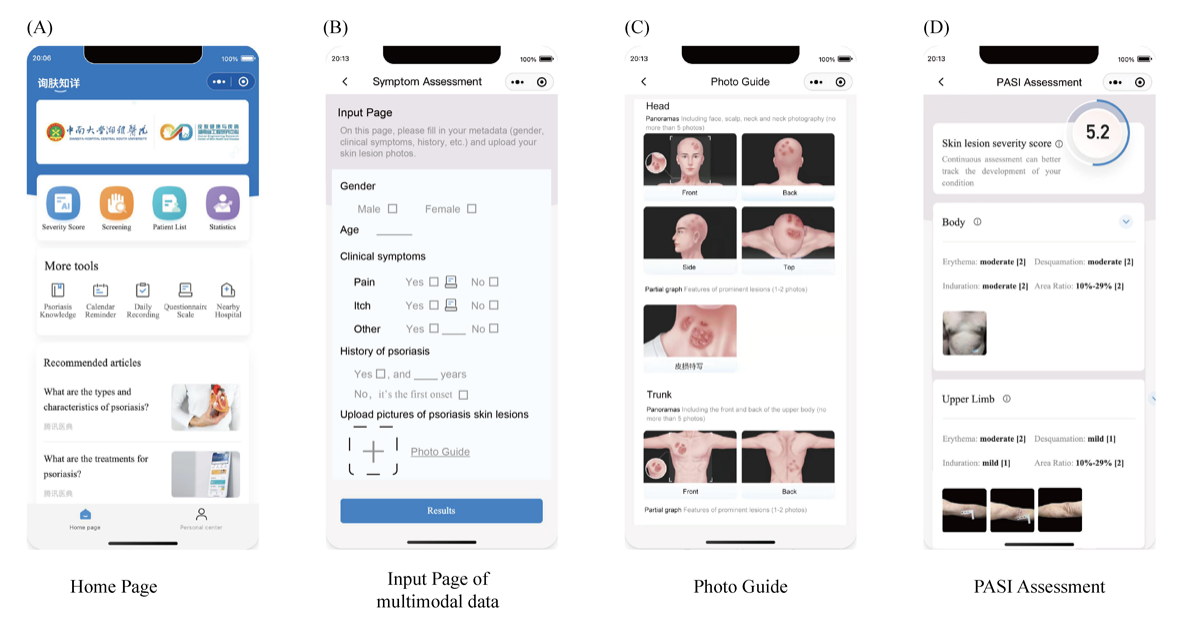
Page introduction and service module of the mobile app SkinTeller. (A) On the first page, mobile app SkinTeller includes the function of severity score (severity rating and psoriasis diagnosis), screening (intelligent diagnosis), patient list (managing the patients who the current dermatologist is response of), statistics (providing data analysis of patients who the current dermatologist is response of), psoriasis knowledge (recommending the articles of psoriasis self-management), calendar reminder (reminding the patients of hospital revisits and medicine dosage), daily recording (recording the vital signs, such as blood pressure, pulse and the medical dosage history), questionnaire scale (Self-rating Anxiety Scale, Self-rating Depression Scale, Health-Related Quality of Life, etc) and nearly hospitals (providing information about nearby hospitals to facilitate patient treatment). (B) On the second page, multimodal data input page (including metadata and images). (C) On the third page, the example of photo guide which instruct the patient how to take pictures of each part of the body. (D) on the last page, the result page includes the overall PASI score as well as all 16 subscores. PASI: Psoriasis Area and Severity Index.

The proposed severity assessment model was deployed as an app on the WeChat platform. Functions and the user interface are shown in Figure 4. Relying on physician resources and high-quality algorithms, the SkinTeller app provides the function of AI-driven diagnosis, generic clinical science knowledge, health consultation, and recommendations to suitable hospitals and doctors. Doctors can also conduct follow-ups on the web. Patients can easily access the specialist-level services, and doctors in primary hospitals might also reach the clinical level of domain experts (Figure 4).

Furthermore, 43 dermatologists from different levels of hospitals were interviewed about SkinTeller (Multimedia Appendix 5). In all, 91% (39/43) of dermatologists perceived SkinTeller as helpful, relieving them from tedious and error-prone PASI scoring, especially dermatologists from country or community hospitals. Additionally, 86% (37/43) considered the SkinTeller app to have efficiently and accurately rated the degree of psoriasis severity. Regarding the management tool for chronic disease, 72% (31/43) confirmed that it might assist them in arranging the re-examination schedule and reminding their patients automatically. Almost all dermatologists (42/43, 98%) were willing to recommend the SkinTeller to colleagues (Table 5).

Regarding potential disadvantages of SkinTeller, 88% (38/43) of dermatologists recommended further improvements by providing alternative treatment options and predicting the possible patient outcomes for the doctor.

**Table 5:** Feedback from 43 dermatologists on the use of the SkinTeller mobile app.

Question, response		Dermatologist (N=43), n (%)	
**Do you think the mini-app is of great help to the diagnosis and treatment of psoriasis?**			
	Very helpful		12 (28)
	A lot helpful		27 (63)
	Generally helpful		4 (9)
**Would you recommend to other doctors or patients the use of the mini-app?**			
	Actively recommend		21 (49)
	Recommend		21 (49)
	It depends		1 (2)
**What do you think is the advantage of the mini-app?**			
	Quickly and accurately assesses the condition of psoriasis		37 (86)
	Provides follow-up plans of patients for doctors and has a function to remind patients		31 (72)
	Can better guide treatment		30 (70)
	Can better judge the prognosis		25 (58)
**After you have used the mini-app, what aspects need to be improved?**			
	The patient cannot cooperate		19 (44)
	Inconvenient operation		17 (40)
	The accuracy is not high		11 (26)
	Unreasonable application scenarios		6 (14)
	Others		2 (5)

## Discussion

### Principal Findings

To solve the problem of PASI scoring in the real clinical practice, an image-AI–based system was developed based on a large multicenter database of patients with psoriasis. In this system, a multiview deep feature enhancement block to combine features from multiple perspectives was designed to simulate the clinical process of PASI calculation as dermatologists. The proposed model outperforms the average performance of 43 experienced dermatologists. Perhaps most importantly, we deployed a mobile app, SkinTeller, integrated with the model, which has been used 3369 times for PASI scoring in 1497 patients from 18 hospitals, and its excellent performance was confirmed by a feedback survey of 43 dermatologist users. This study may provide a promising alternative for accurate assessment and self-management of psoriasis.

About dilemmas in PASI, we conducted a multicenter questionnaire-based investigation among 346 dermatologists (Multimedia Appendix 2). Only 30.9% dermatologists preferred to evaluate the severity of psoriasis by scales. Two major disadvantages of PASI were recognized—having difficulties in calculating the score and the subjectivity of results—which made the evaluating procedure time-consuming and results unreliable. The shortcomings of each part of PASI were probably the sources of prejudice by the doctors. Our solution potentially overcomes the shortcomings of PASI and augments the clinical workflow of psoriasis management. First, SkinTeller integrated with the proposed image-AI–based severity assessment model provides the function of intelligent psoriasis severity rating and recommends personalized treatment. Second, an effective chronic disease management tool in the SkinTeller assists in tracking the condition. Third, under the support of physical hospitals and the construction of internet hospitals, multiple rounds of interactive self-inspection systems and intelligent referral and triage systems are formed.

Prior to our study, Schaap et al [15] constructed a system for automating PASI scores. Fink et al [16] proposed a filter-based image processing pipeline to determine the PASI score, but a limitation is the nonstandardized body posture of patients with immobility or noncooperation when collecting images. In our study, we have established a multicenter collaboration system for psoriasis data collection and formed a psoriasis data collection standard. The real data from patients with psoriasis that had the blur and occlusion removed were used to establish the model, which is fundamental to our study. First, we collected data from 21,524 patients with psoriasis with 5-20 clinical images per patient on average, resulting in the large sample size of the database. Recent studies reported the performance of an image-based automated deep learning system trained using 1731 images [15], and the observer-independent assessment system of psoriasis-affected areas included a total sample of only 259 photographs [20]. Second, the reference value of the PASI was calculated by 3 doctors independently with the supervision of 3 senior doctors, which ensures that the value is reliable. Of course, there are the other score systems such as Physician Global Assessment (PGA) and Body Surface Area (BSA) that are also commonly used in clinic. These scoring methods have different emphases and evaluation indexes. Our system is trained and developed based on PASI score data and outperforms those of dermatologists. However, after changing the task to PGA or BSA scores, the performance can be reduced because the training is inconsistent with the task. In the future, we will try other score methods, including PGA score, BSA score, etc, for psoriasis and strive to upgrade the system based on existing technologies [22] for most scenarios.

Our team has long been researching the intelligent diagnosis and treatment of psoriasis [23]. Besides the large-scale psoriasis database mentioned above, we also proposed a deep learning system to estimate the overall severity score and all intermediate metrics. In a novel approach to model construction [24], skin lesions were first mapped into a special-designed color space and then classified into 3 different types [25]; erythema scores were scored with the K-nearest neighbor algorithm [26], and a Gaussian mixture model was applied to segment skin lesions into different color channels and then scored based on the trichromatic bands. For CNN-based approaches, one model [27] estimates the severity levels of 3 indicators (erythema, induration, and desquamation) by adding 3 output heads in a single deep neural network [28]. These existing studies mainly focus on a partial list of aspects related to the severity of psoriasis and only generate severity levels with 5 discrete severity categories. They do not meet the clinical needs of psoriasis treatment, which requires an overall severity level for convenience and a larger and finer score range to monitor the severity in a timely and precise manner. If the patient’s PASI score decreases after treatment, then the management plan is effective; otherwise, the management plan must be adjusted. In this paper, we proposed to quantitatively estimate the overall severity score and all intermediate metrics. This method makes the psoriasis severity rating more convenient and consistent, especially when the difference in scores between 2 visits is larger than 5, indicating that the model showed clinically satisfying consistency in patients with psoriasis with substantial disease changes. Second, in the clinical diagnosis of psoriasis, multiple perspectives can bring more information to dermatologists. Therefore, to approximate the diagnosis scenario, we designed a multiview feature enhancement block, which integrates features from multiple input images to combine visual features from different perspectives. Third, our system has been comprehensively verified by comparison with doctors, ablation experiments, questionnaire feedback, and so on. In the ablation experiment, 3 input images were considered to have the smallest MAE. Lastly, our system can also assist doctors in making treatment decisions. On this basis, we also developed an app for the whole course management. This system will become a reliable reference for dermatologists to manage patients, predict progression in the long term, and avoid limitations in the evaluation results caused by the subjective judgment of clinicians.

Our study also has limitations. First, our clinical data set was limited to patients of Chinese origin, which would require retraining with local pictures if our approach were to be established elsewhere. Skin color would be expected to have some influence on the accuracy of the PASI score, especially on the subscore of erythema. Second, the data we used were collected from many institutions, which is conducive to the more general applicability of the development results but may also include bias by the different devices used for photography, environmental factors, and so on. Third, future work will be required to develop more user-friendly functions on SkinTeller. We also call for more efforts to assess the impact of this model and resulting software on clinical practice.

### Conclusions

The proposed image-AI–based system can provide more objective and accurate assessment for severity of patients with psoriasis and outperforms experienced dermatologists. It can also predict the direction of severity progress with different ranges of PASI scores precisely. The SkinTeller app integrated with the proposed system show enormous potentialities for precise personalized treatment and chronic disease self-management in patients with psoriasis.

## Appendix

Multimedia Appendix 1 Definition of the Psoriasis Area and Severity Index (PASI) scoring.

Multimedia Appendix 2 Questionnaire on the severity assessment of patients with psoriasis.

Multimedia Appendix 3 Interagreement of dermatologists.

Multimedia Appendix 4 The other case study.

Multimedia Appendix 5 Questionnaire on the use of the app (named SkinTeller).

## Abbreviations

AI: artificial intelligence
BSA: Body Surface Area
CNN: convolutional neural network
MAE: mean absolute error
PASI: Psoriasis Area and Severity Index
PGA: Physician Global Assessment
ROI: region of interest
