# Supplementary material for: Artificial Intelligence–Based Psoriasis Severity Assessment: Real-world Study and Application
Source: J Med Internet Res. 2023 Mar 16;25:e44932. doi: 10.2196/44932 (PMC10131673; doi:10.2196/44932)
Supplement: Multimedia Appendix 1 [file jmir_v25i1e44932_app1.docx]

**Multimedia Appendix 1: Definition of the PASI Scoring.**

Currently in clinical practice, Psoriasis Area and Severity Index (PASI) is the most frequently used indicator. For a patient, the calculation process of PASI is as follows:

1. Dermatologists examine skin lesions from four different body parts of a patient, including head, trunk, upper limbs, and lower limbs.
2. For each body part, dermatologists use three metrics to evaluate the severity of Psoriasis: the redness of erythema, the thickness of induration and the scaling of desquamation. Each metric is represented by an integer score ranging from 0 to 4. The higher the score, the more severe the condition.
3. Then for each body part, dermatologists manually estimate the proportion of skin lesion area to normal skin area, resulting in a corresponding area ratio metric (range from 0 to 6 for 0% to 100%).
4. After acquiring four metrics for four body parts, the overall PASI score can be calculated via Eq.([1](#_bookmark1)) and Eq.(2)

$\mathrm{PASI}_{\mathrm{part}_{i}}=(S_{\mathrm{ery}_{\mathrm{part}_{i}}}+S_{\mathrm{ind}_{\mathrm{part}_{i}}}+S_{\mathrm{des}_{\mathrm{part}_{i}}})\times A_{\mathrm{part}_{i}}$ (1)

$\mathrm{PASI}_{\mathrm{patient}}=\sum_{i}^{\mathrm{parts}} W_{\mathrm{part}_{i}}\times\mathrm{PASI}_{\mathrm{part}_{i}}$ (2)

where $\mathrm{part}_{i}$∈{head, trunk, upper limbs, lower limbs}denotes the ith body part;$W_{\mathrm{part}_{i}}$∈{0.1, 0.3, 0.2, 0.4}denotes the corresponding weight; $S_{\mathrm{ery}_{\mathrm{part}_{i}}}$, $S_{\mathrm{ind}_{\mathrm{part}_{i}}}$, and $S_{\mathrm{des}_{\mathrm{part}_{i}}}$denote the severity scores for the redness of erythema, the thickness of induration, and the scaling of desquamation, respectively, and $A_{\mathrm{part}_{i}}$ denotes the proportion score. Clinically, $S_{\mathrm{ery}_{\mathrm{part}_{i}}}$, $S_{\mathrm{ind}_{\mathrm{part}_{i}}}$, and $S_{\mathrm{des}_{\mathrm{part}_{i}}}$ range from 0 to 4; $A_{\mathrm{part}_{i}}$ ranges from 0 to 6; and $\mathrm{PASI}_{\mathrm{patient}}$ ranges from 0 to 72 [1].

Such a scoring process has two drawbacks. First, dermatologists have to estimate 4 scores $S_{\mathrm{ery}_{\mathrm{part}_{i}}}$, $S_{\mathrm{ind}_{\mathrm{part}_{i}}}$, $S_{\mathrm{des}_{\mathrm{part}_{i}}}$and $A_{\mathrm{part}_{i}}$ for 4 body parts, that is 16 scores in total. The estimation highly depends on the experience and expertise of dermatologists. Different dermatologists or even the same dermatologist could come up with varied score for the same case. Therefore, manual PASI estimation may generate inconsistent scores. For patients whose severity needs to be tracked overtime, inconsistent measuring could mislead the judgment in Psoriasis severity progresses [2] and result in the adoption of an inappropriate therapy.

In addition, since there are 16 scores to be estimated, the calculation process of PASI is quite time-consuming. An experienced dermatologist may need 30 minutes to calculate PASI for a single patient [3]. Considering the large number of psoriasis patients, calculating PASI is a heavy burden for dermatologists.

References:

1. George, Y., Aldeen, M. & Garnavi, R. Automatic psoriasis lesion segmentation in two-dimensional skin images using multiscale superpixel clustering. J. Med. Imaging (2017).
2. Fink, C., Fuchs, T., Enk, A. & Haenssle, H. A. Design of an algorithm for automated, computer-guided pasi measurements by digital image analysis. J. Med. Syst. (2018).
3. Berth-Jones, J. et al. A study examining inter-and intrarater reliability of three scales for measuring severity of psoriasis:Psoriasis area and severity index, physician’s global assessment and lattice system physician’s global assessment. Br. J.Dermatol. 155, 707–713 (2006).
