# Supplementary material for: Artificial Intelligence–Based Psoriasis Severity Assessment: Real-world Study and Application
Source: J Med Internet Res. 2023 Mar 16;25:e44932. doi: 10.2196/44932 (PMC10131673; doi:10.2196/44932)
Supplement: Multimedia Appendix 2 [file jmir_v25i1e44932_app2.docx]

**Appendix 2: Questionnaire on the severity assessment of psoriasis patients**

We invite 346 dermatologists to participate a questionnaire on psoriasis severity assessment. The questionnaire enquires dermatologists about their background, their options towards current Psoriasis treatment, how they evaluate the severity degree, etc. Among all 346 dermatologists, 32 are from community level hospitals, 103 from county level, 124 from city level and 87 from province level. We can ﬁnd even for dermatologists from province-level hospitals, only less than half use PASI to measure psoriasis severity, while others rely on roughly estimation or let patients themselves to decide, which is quite error-prone and inconsistent. PASI adoption rate is even lower in the community-level, county-level and city-level hospitals. We can also find with the increase of hospital level, the PASI adoption rate increases accordingly. This is because for high level hospitals with more experienced dermatologists, they are more likely to use PASI in Psoriasis Diagnosis and treatment which proves the clinic value of PASI from one perspective.

To investigate the significance of PASI in dermatological practice, we conducted a multi-centric questionnaire-based investigation among 346 dermatologists and the results were showed (**Figure S1**). The percentage of rough estimating patients’ condition by experience, which was of relative high inaccuracy, significantly went beyond other methods. However, there were only 30.9% dermatologists preferred to evaluate the severity of psoriasis by quantitative scales. Two major disadvantages of PASI were recognized as having difficulties in calculating and the subjectivity of results, which made the evaluating procedure time-consuming and the results unreliable respectively. The shortcomings of each part of PASI were probably the sources of prejudice against PASI by the doctors. Based on the results above, it was significant to overcome the shortcomings of PASI for improving its usage.

1. Which grade level of your hospital?

A. Community-level hospital B. County-level hospital C. City-level Hospital D. Province-level hospital

2. What is your specialty?

A. Dermatology B. General C. Other departments

3. Your job title?

A. Primary title such as resident doctor B. Middle title such as attending doc- tor C. Senior titles such as director or assistant director physician D. Other

4. Does your hospital has specialist dermatologist?

A. Yes, has only 1 B. Yes, has more than 2 C. No

5. Do you know that psoriasis is a disease that requires long-term follow-up and regular subsequent visits?

A. Don’t know B. Have a little understanding, know basic medical examinations, hormones and other treatments C. Have a better understanding and know basic medication and long-term follow-up treatment, etc.

6. What do you think is the biggest problem with the current treatment of psoriasis? (multiple answers applicable)

A. The cause of disease is unclear B. The patient is not sufficiently aware of the disease C. Low patient follow-up rate D. High treatment costs E. Others

7. In your hospital, the approximate ratio of psoriasis patients with psoriasis on time follow-up or re-examination is?

A. More than 50% B. 30-50% C. Less than 30% D. Don’t know

8. Do you think the reason why patients with psoriasis did not follow up or re-examination on time is? (multiple answers applicable)

A. Patients do not pay attention to follow-up/re-examination B. The patient is not sufficiently aware of the disease C. Poor drug reactivity D. The patient does not trust the doctor E. The follow-up/re-examination process is long, time-consuming, and too cumbersome F. Others, specifically

9. When assessing the severity of psoriasis, which method would you choose for assessment?

A. Calculate PASI, BSA, PGA scores, etc. B. Roughly estimate the improve- ment of the patient’s skin lesions C. Patient’s self-description D. Don’t know

10. The PASI score is an internationally recommended method for evaluating the severity of psoriasis. In your opinion, the main deficiencies of this score are? (multiple answers applicable)

A. No shortcomings, very applicable B. The calculation is cumbersome and time-consuming C. Affected by the subjectivity of the evaluator D. The results are rough, with poor repeatability and sensitivity E. Others

11. In your opinion, for the long-term management of patients with psoriasis, which of the following needs to be improved? (multiple answers applicable)

A. Timely diagnosis, disease evaluation and treatment solution optimization B. Doctor-patient communication and health knowledge education C. Regular follow-up and treatment process optimization for patients D. Establish a follow-up medical team E. Others


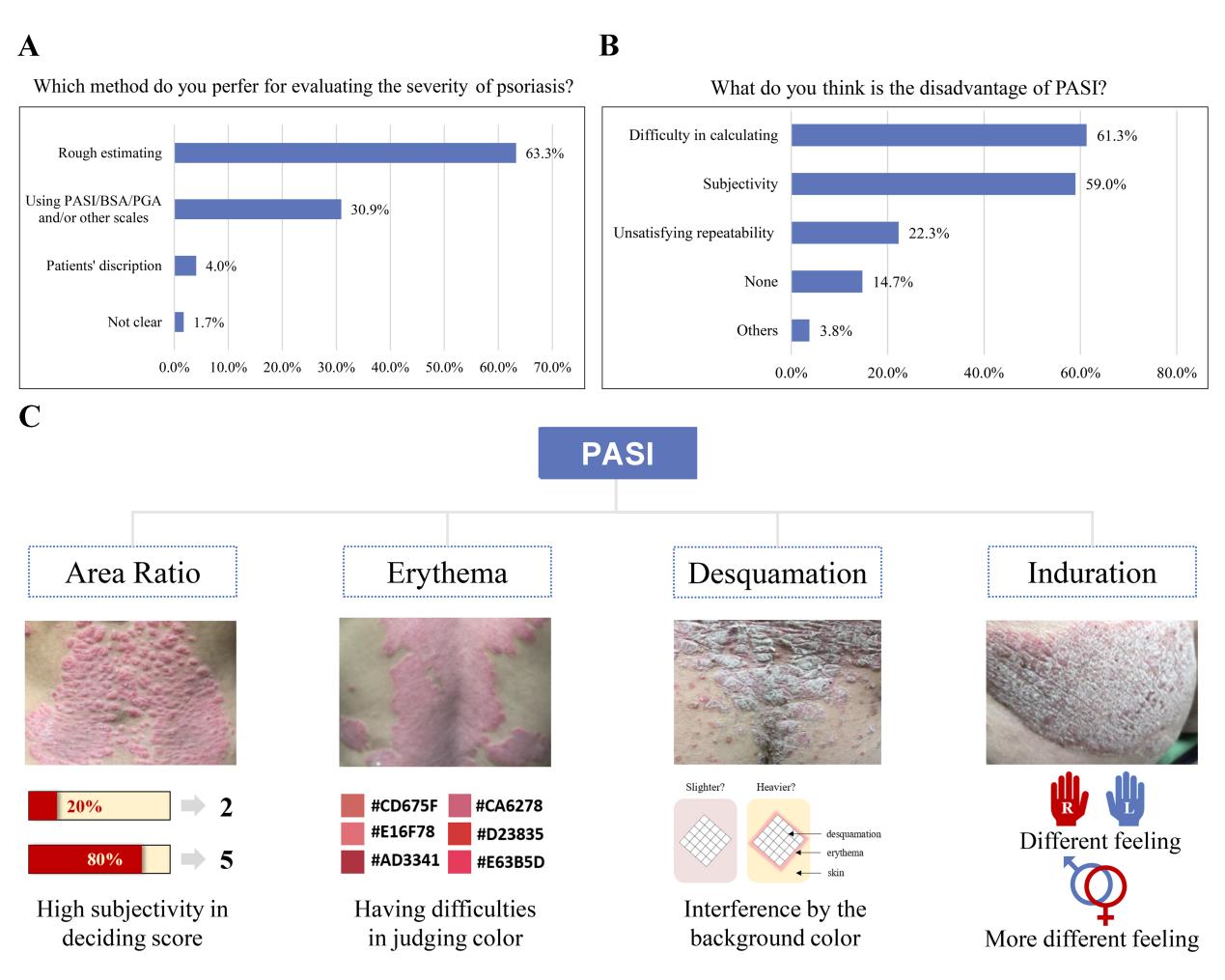


**Figure S1. The importance and shortcoming of the existing PASI method.** (A,B) The results of a multi-central questionnaire among 346 dermatologists. (C) The shortcoming of each part of PASI scale.
