# Supplementary material for: Artificial Intelligence–Based Psoriasis Severity Assessment: Real-world Study and Application
Source: J Med Internet Res. 2023 Mar 16;25:e44932. doi: 10.2196/44932 (PMC10131673; doi:10.2196/44932)
Supplement: Multimedia Appendix 3 [file jmir_v25i1e44932_app3.docx]

**Appendix 3: Inter-Agreement of dermatologists**

We also evaluate the inner-agreement between these 43 dermatologists. We enumerate all pairs of 43 dermatologists and calculate the average Fleiss’s kappa, that is 0.174 for erythema, 0.155 for induration, 0.173 for desquamation and 0.170 for area ratio. The dermatologists don’t align quite well, this is may due to that scoring severity degree is quite subjective, even for experienced dermatologists, they may still be difficult to reach an agreement. Another possibility is that the dermatologists only review the skin lesion pictures instead of scoring in a face to face manner. We use the function defined in Eq.(3) to measure the agreement between two dermatologists. The denominator is the total sub scoring of 13 patients is 13*16=208, and the nominator is number of the sub scores these two dermatologists are the same. The Agreement function ranges from 0 to 1, the larger this value is, the more these two dermatologists agree with each other. We rank the dermatologists according to their PASI scoring accuracy and visualize the agreements between any two of them in **Figure S2**.

$Aggrement\left( D_{1},D_{2} \right)=\frac{\#of same sub scoring}{\#of total sub scoring}$ Eq.(3)

In Figure S3, we use lighter color to denote high agreement and use heavy color to denote low agreement. As a result, the color of cubic in the diagonal Figure S3 is white. As one is agree perfectly with himself. And for top ranked dermatologists, they tend to better agree with each other; For low ranked dermatologists, they’re more likely to disagree with each other.


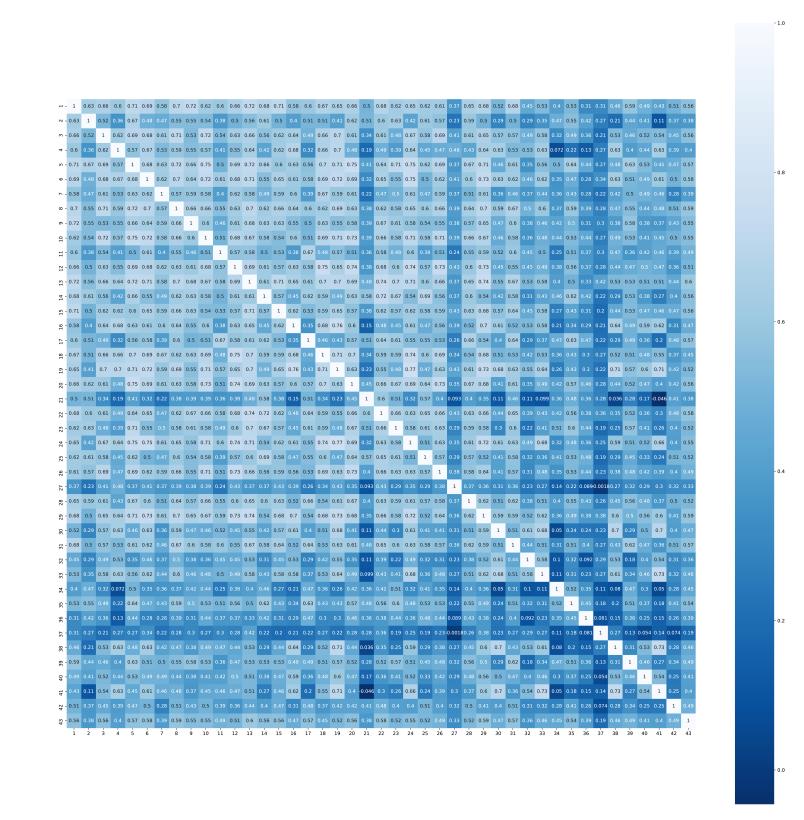


**Figure S2.** **The Agreement among all 43 Dermatologists.** Light color denotes high correlations and dark color denotes low correlations.
