# Supplementary material for: Artificial Intelligence–Based Psoriasis Severity Assessment: Real-world Study and Application
Source: J Med Internet Res. 2023 Mar 16;25:e44932. doi: 10.2196/44932 (PMC10131673; doi:10.2196/44932)
Supplement: Multimedia Appendix 4 [file jmir_v25i1e44932_app4.docx]

**Appendix 4: Case study**

We track a complete treatment course of a psoriasis patient in Xiangya Hospital. Two senior dermatologists join this study. In different treatment phase, they evaluate the severity scores separately, compared with our AI method. The Figure S3A shows the PASI scores of the AI and the doctors at different treatment phases. Although there are some little gaps between the scores, the overall trends match very well.

***Origin Status.***

The Figure S3B shows the origin status of this patient. There are large areas of erythema and desquamation all over the patient, and the thickness of the induration is also very serious, so most of the sub-scores are 3 or 4. Especially in the lower limbs, the area of the skin lesions is very large, basically covering the hips and legs, so the AI and the dermatologists’ scores are both 4. Overall, the patient’s condition is still relatively serious, so our AI algorithm and the two dermatologists’ PASI scores for the patient are both 20+. The MAE between the AI’s PASI score (28.8) and two doctors’ average PASI score (26.95) is 1.85, and the difference is 6.86%.

***Treatment Phase 1.***

The treatment works well from the photos. The head has no skin lesions, so both AI and doctors rate all 0 scores. The area ratio of the skin lesions on the trunk and upper limbs are still not very small, but the severity of the erythema, induration and desquamation has been relatively low. The condition of the lower limbs is still a bit serious, mainly the area ratio and erythema, so AI and doctors still give the relatively high scores, respectively (3, 3, 4) and (2, 2, 2).

***Treatment Phase 2.***

After two phases of treatment, the patient’s condition has been greatly improved. This improvement can be told from the patient photos and the doctors’ scores. The skin lesion is almost disappeared from the head, trunk and the upper limbs, and both AI and doctor.1 consider the illness is completely recovered at those parts and rate all 0 scores. Only a few erythema and desquamation on the lower lambs, and prediction difference from the AI and doctors at this part is the area ratio scores (2,1,1). But compared with Phase 1 (3,3,4), they all think the severity of the area ratio declines.


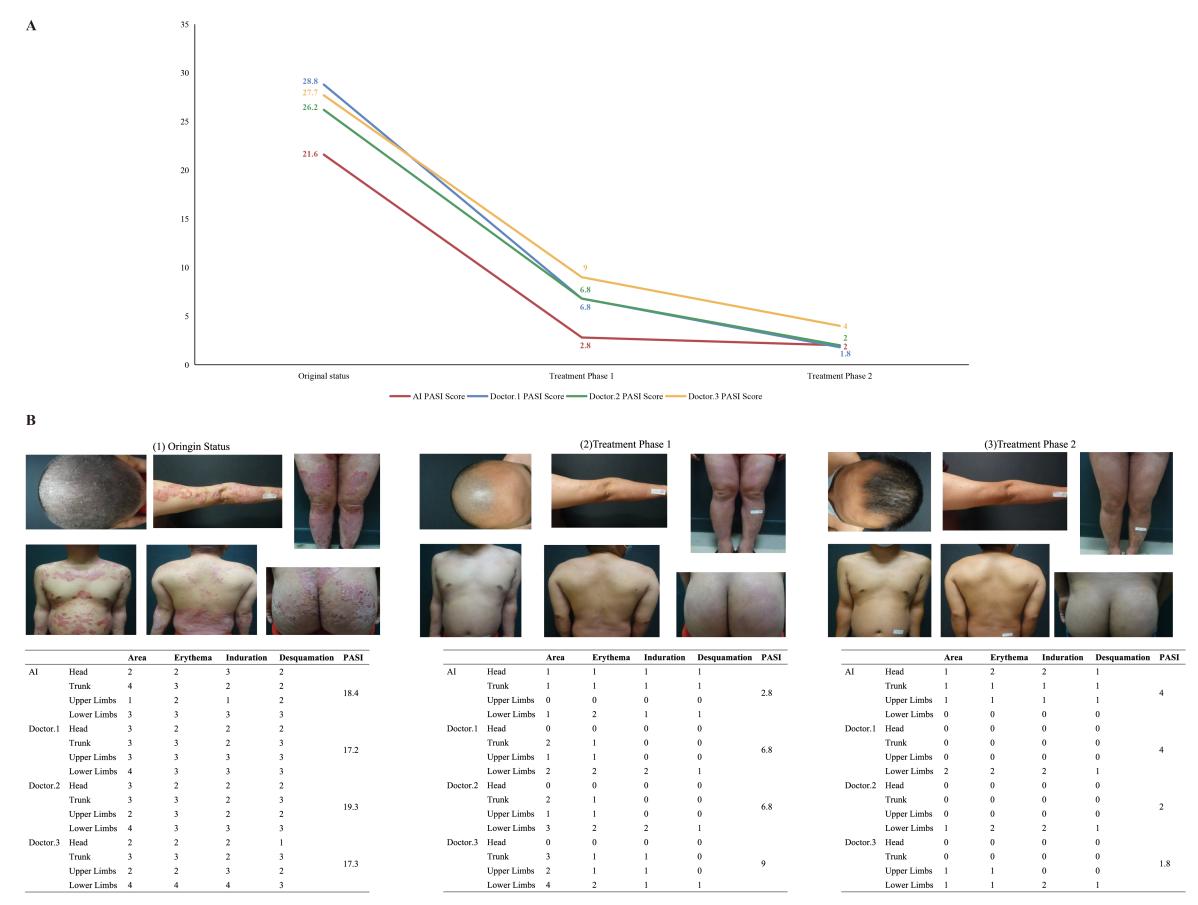


**Figure S3.** The patient status and the PASI scores of another patient between AI and Dermatologists at different treatment phases.
