# Supplementary material for: Artificial Intelligence–Based Psoriasis Severity Assessment: Real-world Study and Application
Source: J Med Internet Res. 2023 Mar 16;25:e44932. doi: 10.2196/44932 (PMC10131673; doi:10.2196/44932)
Supplement: Multimedia Appendix 5 [file jmir_v25i1e44932_app5.docx]

**Appendix 5: Questionnaire on the Usage of the APP (named SkinTeller)**

1. Which level of hospital are you a doctor?

A. Township hospital or community hospital B. County hospitals C. City Hospital D. Provincial and ministerial hospitals

2. What is your specialty?

A. Dermatology B. General C. Other departments

3. Your job title?

A. Primary title such as resident doctor B. middle title such as attending doc- tor C. Senior titles such as director and assistant director physician D. Other

4. Do you think that the APP products of psoriasis is meaningful for diagnosing and evaluating the condition of psoriasis?

A. Doesn’t make sense B. It has a little significance, it can assist the primary doctors C. It is significant and can help the primary doctors well D. It is of great significance and can play a vital role, replacing the doctor in some aspects E. Can completely replace the doctors

5. Do you think that the SkinTeller APP is of great help to the diagnosis and treatment of psoriasis?

A. Very helpful B. A lot helpful C. General helpful D. Little helpful

6. What do you think is the advantage of the SkinTeller APP? (multiple answers applicable)

A. Can better guide treatment B. Quickly and accurately assess the condition of psoriasis C. Provides follow-up plans of patients for doctors and has a function to remind patients D. Can better judge the prognosis E. Others

7. After you have used the SkinTeller APP, what aspects need to be improved? (multiple answers applicable)

A. Inconvenient operation B. The accuracy is not high C. Unreasonable application scenarios D. The patient cannot cooperate E. Others

8. Do you think the SkinTeller APP can be a recommended application?

A. Impossible, there is still a lot of gap with medical practice B. It is possible, but it will take time C. Hopeful, sure to be realized, clinical application

9. Would you recommend to other doctors or patients to use the SkinTeller APP?

A. Actively recommends B. Recommend C. It depends D. Not recommended

10. In the future, in what ways do you hope that the SkinTeller APP can help you? (multiple answers applicable)

A. Auxiliary diagnosis, improve psoriasis classiﬁcation performance B. Before the doctor choose the treatment solution, it proposes several alternatives and predicts the possible results for the doctor C. When the doctor determines the treatment solution or analyzes the treatment condition of the follow-up patients, it evaluates and makes recommendations for modification, and predicts possible results D. Accurately assess the condition of patients with psoriasis, predict disease progression and outcome or the probability and time of complications occur E. Classify and analyze follow-up data of all patients of the same type over a period of time for doctors and provide reports F. Others.
